# Silent Inflammation, Loud Consequences: Decoding NLR Across Renal, Cardiovascular and Metabolic Disorders

**DOI:** 10.3390/ijms26178256

**Published:** 2025-08-26

**Authors:** Caterina Carollo, Alessandra Sorce, Emanuele Cirafici, Maria Elena Ciuppa, Giuseppe Mulè, Gregorio Caimi

**Affiliations:** Department of Health Promotion, Mother and Child Care, Internal and Specialistic Medicine, University of Palermo, 90127 Palermo, Italy; alessandra.sorce@community.unipa.it (A.S.); emanuele.cirafici@community.unipa.it (E.C.); mariaelena.ciuppa@community.unipa.it (M.E.C.); giuseppe.mule@unipa.it (G.M.); gregorio.caimi@unipa.it (G.C.)

**Keywords:** NLR, inflammation, CKD, hypertension, diabetes, cardiovascular disease

## Abstract

The neutrophil-to-lymphocyte ratio (NLR) has emerged as a readily accessible, cost-effective biomarker reflecting systemic inflammation. Chronic low-grade inflammation plays a pivotal role in the pathogenesis and progression of metabolic and cardiovascular disorders including chronic kidney disease (CKD), hypertension, diabetes mellitus, and cardiovascular disease (CVD). This review critically evaluates the current evidence on NLR as a prognostic marker across these interconnected conditions. A comprehensive literature search was conducted focusing on clinical and epidemiological studies investigating the association between NLR and CKD, hypertension, diabetes, and cardiovascular outcomes. Mechanistic insights into inflammation-driven pathophysiology and the predictive utility of NLR in disease progression and adverse events were synthesized. Elevated NLR is consistently associated with increased risk and severity of CKD, correlating with glomerular filtration decline, proteinuria, and mortality. In hypertension, higher NLR levels are linked to non-dipper blood pressure patterns, arterial stiffness, and increased cardiovascular risk. Among diabetic patients, NLR correlates with poor glycemic control and vascular complications. In cardiovascular disease, elevated NLR predicts major adverse cardiovascular events (MACE) and all-cause mortality, reflecting underlying immune dysregulation and endothelial dysfunction. Despite promising findings, direct comparisons with established inflammatory biomarkers remain limited, and heterogeneity exists across populations. NLR represents a simple yet powerful inflammatory biomarker with significant prognostic value in CKD, hypertension, diabetes, and cardiovascular disease. Its integration into clinical risk stratification models could enhance personalized medicine approaches. Future research should focus on longitudinal studies, validation in diverse cohorts, and comparative analyses with other inflammatory markers to fully delineate NLR’s clinical utility.

## 1. Introduction

Chronic low-grade inflammation, often termed “silent” or subclinical inflammation, represents a fundamental pathophysiological process underpinning a range of chronic diseases, including chronic kidney disease (CKD), diabetes mellitus, cardiovascular disease (CVD), and hypertension [1,2,3]. Unlike overt inflammatory conditions characterized by acute immune activation and clinical symptoms, silent inflammation is a persistent, low-intensity immune response that progressively contributes to tissue injury and organ dysfunction over time [4]. In CKD, silent inflammation accelerates renal fibrosis and functional decline through sustained activation of innate immune cells and pro-inflammatory cytokines [5,6]. Similarly, in diabetes mellitus, chronic subclinical inflammation is implicated in insulin resistance, β-cell dysfunction, and the development of microvascular and macrovascular complications [7]. Cardiovascular disease is driven, in part, by inflammation-mediated endothelial dysfunction, atherosclerotic plaque formation, and destabilization [8,9]. Hypertension itself is increasingly recognized as an inflammatory disorder, where immune activation promotes vascular remodeling and stiffness [9,10]. This shared inflammatory milieu establishes silent inflammation as a unifying mechanism that links metabolic, renal, and cardiovascular pathologies, highlighting the need for biomarkers and therapeutic strategies targeting this covert immune dysregulation.

The neutrophil-to-lymphocyte ratio (NLR) has gained considerable attention as a readily available biomarker reflecting systemic inflammation. Derived from routine complete blood counts, NLR offers a simple, cost-effective, and widely accessible measure that integrates innate and adaptive immune system components [11,12]. Neutrophils, key mediators of acute and chronic inflammatory responses, tend to increase during systemic inflammation [4], whereas lymphocytes often decrease in settings of physiological stress and immune dysregulation [13]. This dynamic shift results in an elevated NLR, which correlates with the degree of underlying inflammation. Compared to traditional inflammatory markers such as C-reactive protein (CRP) or interleukin-6 (IL-6), NLR benefits from its low cost, rapid availability, and ease of interpretation, making it a practical tool for clinical and research applications across diverse patient populations.

This review aims to comprehensively explore the potential of the neutrophil-to-lymphocyte ratio (NLR) as a versatile biomarker of subclinical inflammation across CKD), diabetes mellitus, cardiovascular disease, and hypertension. By synthesizing existing evidence, we seek to underscore the utility of NLR in risk stratification, disease monitoring, and prognosis within these interconnected conditions. Furthermore, the review highlights the current gaps and limitations in the literature, including inconsistencies in threshold values, mechanistic understanding, and clinical applicability, to guide future research directions and promote standardized use of NLR in clinical practice.

## 2. Materials and Methods

This work is a narrative review aiming to synthesize current evidence on the role of NLR as a marker of immune dysregulation and inflammation in the context of diabetes mellitus, CKD, and cardiovascular disease, with a focus on shared pathophysiological mechanisms such as endothelial dysfunction, oxidative stress, and systemic immune imbalance. A non-systematic, yet comprehensive literature search was conducted using PubMed, Web of Science and Google Scholar in May 2025. The following keywords and Boolean operators were used to retrieve relevant publications: (“neutrophil-to-lymphocyte ratio” OR NLR) AND (diabetes OR “chronic kidney disease” OR CKD OR “cardiovascular disease” OR CVD).


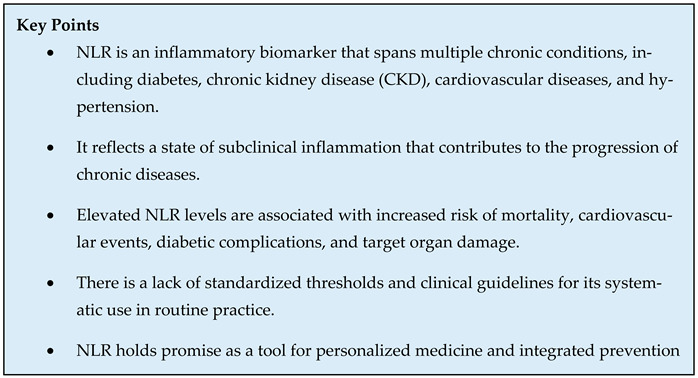


We included original research articles, meta-analyses, and narrative or systematic reviews published up to 1 June 2025, without language or publication date restrictions. Priority was given to studies reporting on human subjects and discussing pathophysiological mechanisms linking NLR with one or more of the four conditions (diabetes, CKD, hypertension and CVD), either separately or in combination. Studies were selected based on their relevance to the review’s aims, methodological rigor, and contribution to understanding shared inflammatory and immune-mediated pathways. Given the narrative nature of the review, no formal risk of bias assessment or meta-analysis was performed. Instead, findings were grouped thematically and discussed in relation to key pathophysiological domains: endothelial dysfunction, oxidative stress, and immune dysregulation, with reference to clinical correlates such as disease progression and cardiovascular or renal outcomes.

## 3. NLR and Inflammation: Biological Basis

Neutrophils and lymphocytes represent two fundamental arms of the immune system—innate and adaptive, respectively—but their functions are deeply intertwined in shaping the host inflammatory response. Their recruitment, activation, and effector profiles define not only the initial immune engagement but also the trajectory of chronic inflammation in cardio-renal-metabolic disorders **[14,15]**.

Neutrophils, the most abundant circulating leukocytes, act as the first responders of innate immunity. They are rapidly recruited to sites of infection or tissue injury in response to chemokine gradients and danger-associated molecular patterns **[16]**. Upon activation, neutrophils engage in phagocytosis, degranulation, production of reactive oxygen species (ROS) **[17]**, and release of extracellular traps (NETs) and pro- and anti-inflammatory cytokines **[18]**. While these responses are essential for pathogen clearance, dysregulated neutrophil activation may result in bystander tissue injury and contribute to chronic inflammation. A significant contributor to neutrophil-mediated oxidative stress is the formation of reactive carbonyl species—electrophilic compounds with aldehyde and ketone groups—produced through lipid peroxidation and protein oxidation **[19]**. These reactive carbonyls can modify cellular proteins through carbonylation, altering signaling pathways and exacerbating tissue damage.

Recent insights have redefined neutrophils as modulators of immunity rather than mere terminal effectors. Through cytokine secretion and dynamic cross-talk with dendritic cells and lymphocytes, neutrophils influence the magnitude and quality of adaptive immune responses. They may also carry an “immunological blueprint” that shapes downstream immune cascades **[20,21]**. Sustained neutrophil activation has been implicated in chronic inflammatory states such as atherosclerosis, autoimmunity, cancer, and CVD. Neutrophil death pathways, including NETosis and pyroptosis, further amplify inflammation and may expose autoantigens, perpetuating immune dysregulation **[22]**.

Lymphocytes—primarily T and B cells—form the backbone of adaptive immunity. CD4^+^ T-helper subsets (Th1, Th2, Th17) orchestrate immune responses against specific pathogen classes. Th1 cells drive macrophage activation via interferon-gamma (IFN-γ), Th2 cells promote eosinophilic responses through IL-4, IL-5, and IL-13, and Th17 cells recruit neutrophils through IL-17 and IL-22 **[23]**. Regulatory T cells (Tregs) are crucial for maintaining immune tolerance and suppressing excessive inflammation **[24]**. A decline in circulating lymphocytes—due to apoptosis, sequestration, or exhaustion—is frequently observed in chronic inflammatory conditions such as diabetes and CKD, and is associated with adverse clinical outcomes **[25]**.

Additionally, innate lymphoid cells (ILCs) have emerged as critical players in bridging innate and adaptive immunity. Though antigen-independent, ILCs mirror the cytokine profiles of T-helper subsets. Group 1 ILCs (e.g., NK cells) produce IFN-γ; Group 2 ILCs (e.g., nuocytes) produce IL-5 and IL-13; and Group 3 ILCs produce IL-17 and IL-22, paralleling Th17 responses. These cells contribute to tissue-specific immune responses and may play roles in mucosal defense and chronic inflammation. Their development relies on the transcription factor Id2 and common γ-chain cytokines such as IL-7, indicating shared lineage with classical lymphocytes **[26,27]**.

However, the balance between neutrophils and lymphocytes is also tightly regulated by transcriptional programs that orchestrate inflammatory signaling. Among them, NF-κB, AP-1, STAT-3, and Nrf2 are particularly relevant.

NF-κB and AP-1 act as pro-inflammatory hubs, driving expression of chemokines, adhesion molecules, and survival factors that prolong neutrophil activity, thereby favoring neutrophilia. STAT-3, activated downstream of IL-6 and related cytokines, promotes Th17 differentiation and neutrophil recruitment while contributing to lymphocyte exhaustion. In contrast, Nrf2 provides a counter-regulatory mechanism by inducing antioxidant defenses and limiting oxidative stress; its dysfunction enhances neutrophil-driven damage and accelerates lymphocyte loss. Importantly, redox-sensitive pathways involving these transcription factors can be therapeutically modulated—for instance, polyphenols have been shown to activate Nrf2 and dampen NF-κB/AP-1 signaling, reducing systemic inflammation [28].

The neutrophil-to-lymphocyte ratio (NLR) has emerged as a practical biomarker representing this complex immunological equilibrium. A high NLR reflects a dominance of innate pro-inflammatory responses over adaptive regulatory mechanisms—a hallmark of immune dysregulation and “inflammaging,” the chronic low-grade inflammation observed in aging, metabolic stress, and endothelial dysfunction **[29,30]**. Elevated NLR has been linked to disease progression in hypertension **[31]**, diabetes **[32]**, CKD **[33,34]**, and CVD **[35]**, suggesting its role extends beyond a surrogate of inflammation to a window into pathophysiological processes driving organ-specific injury.

**Figure 1:**
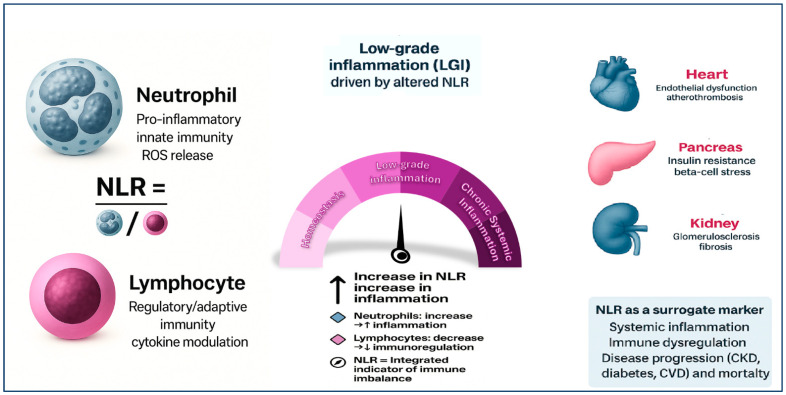
The neutrophil-to-lymphocyte ratio (NLR) as an integrated marker of immune imbalance and chronic low-grade inflammation (LGI). *NLR reflects the balance between pro-inflammatory neutrophils and regulatory lymphocytes, integrating signals from both the innate and adaptive immune systems. An elevated NLR—driven by neutrophilia, lymphopenia, or both—indicates immune dysregulation and promotes a state of low-grade inflammation (LGI), which may evolve into chronic systemic inflammation. This immune imbalance contributes to the pathogenesis of major cardio-renal-metabolic diseases. In hypertension, elevated NLR is associated with endothelial dysfunction and vascular remodeling. In diabetes, it correlates with insulin resistance and β-cell stress. In CKD, increased NLR is linked to glomerulosclerosis, fibrosis, and progression of renal impairment. In CVD, NLR predicts atherogenesis, plaque instability, and adverse cardiovascular outcomes. Therefore, NLR serves as a low-cost, accessible surrogate marker of systemic inflammation, immune activation, and disease progression across multiple chronic conditions*.

In metabolic cardiorenal syndrome—where diabetes, CVD, and CKD coexist in a synergistic and self-amplifying pathophysiological loop—NLR might provide critical information about systemic immune activation. An elevated NLR reflects increased neutrophil-mediated endothelial dysfunction, atherosclerotic instability, and renal fibrosis, coupled with lymphopenia arising from metabolic stress and immune suppression. This imbalance marks a shift toward innate immune predominance, which promotes vascular injury, impairs reparative responses, and accelerates multiorgan deterioration **[36]**. The pathophysiological interplay between CKD, diabetes mellitus, and CVD is underpinned by a constellation of shared mechanisms, notably endothelial dysfunction, oxidative stress, and immune dysregulation **[37]**. Hyperglycemia, uremic toxins, and dyslipidemia synergistically impair endothelial integrity, promoting vascular stiffness and atherogenesis. Concurrently, elevated levels of ROS and systemic inflammation perpetuate oxidative stress, exacerbating tissue injury across the renal, cardiovascular, and metabolic systems **[38]**. Within this context, NLR has emerged as a clinically accessible marker reflecting this pro-inflammatory and immune-altered milieu. Increased neutrophil counts reflect activation of innate immune pathways and oxidative burst activity, while decreased lymphocyte levels may result from chronic metabolic stress and immunosuppressive signaling. This imbalance is particularly relevant in the metabolic cardiorenal continuum, where NLR correlates with endothelial dysfunction, vascular calcification, and adverse clinical outcomes. As such, NLR not only represents a surrogate of systemic inflammation but also integrates key pathophysiological features common to CKD, diabetes, and CVD [14,39] (Figure 1).

## 4. NLR and Diabetes

In individuals with diabetes, chronic low-grade inflammation and immune system dysfunction plays a pivotal role in both disease onset and progression **[40]**. This inflammatory milieu is closely linked to the development of insulin resistance, β-cell dysfunction, and vascular damage **[41]**. Type 2 diabetes frequently develops in the context of obesity, advancing age, and sedentary lifestyle. The disease arises from the progressive inability of pancreatic β-cells to adequately compensate for insulin resistance. Several mechanisms have been proposed to account for impaired insulin action and secretion, including oxidative and endoplasmic reticulum stress, deposition of amyloid within pancreatic islets, abnormal fat accumulation in liver, muscle, and pancreas, as well as lipotoxic and glucotoxic effects [34,35]. These metabolic and cellular disturbances not only trigger inflammatory pathways but are also aggravated by pre-existing inflammation.

Evidence of innate immune activation has been detected in the circulation, insulin-sensitive tissues, and pancreatic islets of individuals with type 2 diabetes, reinforcing the concept that inflammation contributes to disease onset and progression. Proposed drivers of this pro-inflammatory state include adipose tissue expansion with hypoxia and cell death, activation of the nuclear factor-κB (NF-κB) and c-Jun N-terminal kinase (JNK) signaling cascades, stimulation of interleukin-1β (IL-1β), and recruitment of immune cells into metabolic tissues [42]. Clinical studies using anti-inflammatory agents such as IL-1 antagonists or salsalate have provided encouraging results, highlighting inflammation as a promising therapeutic target [43].

A growing body of evidence suggests that inflammatory processes play a significant role in the pathogenesis of type 2 diabetes, although their precise contribution and therapeutic implications are still being clarified. The neutrophil-to-lymphocyte ratio (NLR) has repeatedly been linked to diabetes within this context, with higher NLR values reported in patients compared to healthy controls [44,45,46]. Interestingly, elevated NLR has also been documented in individuals with prediabetes, indicating that immune imbalance may be detectable even at early stages of metabolic dysfunction.

A large prospective study based on NHANES data included 13,024 adults with prediabetes, followed over approximately 8.5 years. Participants were stratified into NLR tertiles (≤1.60, 1.60–2.29, ≥2.29). After adjusting for confounders, each one-unit increase in NLR was associated with a 12% higher risk of all-cause mortality (HR 1.12; 95% CI, 1.05–1.19) and a 24% increase in cardiovascular mortality (HR 1.24; 95% CI, 1.11–1.37) compared to the lowest NLR group. Individuals in the highest NLR tertile showed a significantly increased risk (HR ~1.21 for all-cause mortality, ~1.49 for cardiovascular mortality) compared to those in the lowest tertile **[42]**.

In addition, a 3-year follow-up study on individuals with diabetes and prediabetes found that elevated NLR was an independent predictor of worsening renal function **[43]**.

These findings support a significant prognostic role for NLR even at the early stages of glucose metabolism dysregulation, suggesting its potential utility not only in established diabetes but also in prediabetes, for risk stratification and clinical monitoring.

Most of the existing literature on NLR in the context of diabetes has predominantly focused on its association with glycemic control. Elevated NLR values have been consistently linked to higher HbA1c levels, poor metabolic control, and increased insulin resistance, suggesting that systemic low-grade inflammation plays a role in the deterioration of glycemic homeostasis. A 2023 meta-analysis by Adane et al., which included 13 studies, demonstrated that patients with poor glycemic control exhibited significantly higher NLR values compared to those with adequate control **[44]**. These findings reinforce the association between elevated NLR and increased HbA1c levels in individuals with type 2 diabetes. Consequently, NLR may be considered a complementary marker to HbA1c for assessing glycemic control in T2DM patients.

However, despite this growing body of evidence, relatively few longitudinal or prospective studies have explored the prognostic value of NLR in predicting diabetes-related complications, such as nephropathy, retinopathy, or cardiovascular events. Beyond glycemic control, higher NLR levels have been identified as independent predictors of diabetic nephropathy, retinopathy, and cardiovascular mortality in patients with T2DM.

A recent study using data from the NHANES 2009–2018 cycles investigated the association between inflammatory biomarkers and clinical outcomes in individuals with diabetic retinopathy (DR). The analysis included 572 adults with DR and examined the prognostic value of three inflammation-based indices: the neutrophil-to-lymphocyte ratio (NLR), monocyte-to-lymphocyte ratio (MLR), and the systemic inflammation response index (SIRI). Results showed that elevated levels of these biomarkers were significantly associated with increased all-cause and diabetes-related cardiovascular mortality. Specifically, participants with NLR ≥ 1.516, MLR ≥ 0.309, or SIRI ≥ 0.756 had markedly higher mortality risks compared to those below these thresholds. The associations were even more pronounced in individuals aged 60 years or older. Furthermore, the combined use of NLR, MLR, and SIRI slightly improved predictive accuracy for mortality compared to individual markers alone **[45]**. These findings support the potential role of inflammatory markers as prognostic tools in the diabetic retinopathy population, highlighting the contribution of systemic inflammation to adverse outcomes in this high-risk group. Similar findings were reported by Wang and colleagues, who demonstrated that elevated NLR and PLR are significantly associated with diabetic retinopathy (DR) in patients without a family history of diabetes or hypertension. NLR emerged as an independent risk factor, while high PLR levels were linked to increased DR risk in the upper quantile. The inclusion of NLR and PLR improved DR risk prediction beyond hemoglobin, enhancing model discrimination and reclassification **[46]**

These findings may suggest that NLR may serve not only as a marker of systemic inflammation but also as a prognostic indicator for long-term outcomes in diabetic populations. Additionally, its role in risk stratification and its potential integration into multiparametric models for personalized prevention and treatment strategies remain largely unexplored. This highlights the need for broader investigation beyond glycemic indices to fully elucidate the clinical utility of NLR in diabetes care. Table 1 summarized the main studies reviewed in this section.

## 5. NLR and CKD

The relationship between systemic inflammation CKD has long been recognized, with a growing body of literature indicating that inflammation is not merely a consequence but a central driver of CKD progression. Among inflammatory markers, NLR has emerged as a particularly promising indicator, offering a cost-effective, accessible, and dynamic tool for assessing inflammatory burden in CKD patients.

Multiple studies have documented a progressive increase in NLR across CKD stages, correlating with declining glomerular filtration rate (GFR), increased proteinuria, and worsening histological damage such as interstitial fibrosis and tubular atrophy. In a pivotal prospective study by Yoshitomi et al. **[47]** involving 256 non-dialysis CKD patients followed for nearly three years, elevated baseline NLR was independently associated with a higher risk of renal outcomes, including significant eGFR decline and initiation of dialysis. Notably, these findings remained robust after adjusting for traditional risk factors, underscoring the independent prognostic role of inflammatory activation in renal deterioration.

Other large cohort studies have reinforced these associations. In a study of 966 patients with biopsy-proven IgA nephropathy, those with NLR ≥ 2.67 were significantly more likely to progress to end-stage kidney disease, and elevated NLR was closely linked to histopathologic findings such as glomerulosclerosis and interstitial fibrosis **[48]**.

In a large cross-sectional study involving 2846 apparently healthy adults (1777 men and 1069 women), the association between NLR and CKD was evaluated with a focus on the impact of body weight. Participants were categorized into normal-weight and overweight/obese groups based on BMI criteria. The study found that higher NLR values were significantly associated with increased CKD prevalence in overweight and obese individuals—both in men (adjusted OR = 1.37, 95% CI: 1.03–1.82, *p* = 0.03) and women (adjusted OR = 1.77, 95% CI: 1.08–2.90, *p* = 0.023)—but not in those with normal weight.

Further, in overweight/obese participants with mildly to moderately reduced eGFR (50–70 mL/min/1.73 m^2^), NLR was inversely correlated with eGFR in both sexes, suggesting a link between systemic inflammation and early renal function decline. These findings point to a pathophysiological interplay between adiposity-related low-grade inflammation and renal injury, where elevated NLR may reflect a heightened inflammatory burden that contributes to glomerular damage, endothelial dysfunction, and progressive renal impairment.

The absence of a significant association in normal-weight individuals reinforces the hypothesis that metabolic inflammation—characteristic of obesity and insulin resistance—may potentiate the deleterious renal effects of immune dysregulation. NLR may thus serve as a valuable early indicator of CKD risk in overweight and obese populations **[49]**.

Mechanistically, elevated NLR in CKD appears to reflect the confluence of several pathophysiological processes. Neutrophil activation in renal disease promotes endothelial dysfunction, capillary rarefaction, and the release of reactive oxygen species and neutrophil extracellular traps (NETs), all of which accelerate glomerular and tubulointerstitial injury. In contrast, lymphopenia reflects impaired regulatory immune responses and reduced capacity to resolve inflammation, creating a chronic pro-inflammatory state that fuels fibrosis. This dysregulated immunological environment fosters progression of CKD and increases susceptibility to cardiovascular events, a leading cause of death in this population.

The prognostic relevance of NLR extends to patients undergoing renal replacement therapy. In a meta-analysis encompassing over 116,000 CKD and dialysis patients, high NLR was significantly associated with increased all-cause and cardiovascular mortality **[50]**. In a Romanian end-stage renal disease cohort, individuals in the high-NLR group exhibited a 30-day mortality rate exceeding 40%, compared to less than 2% among those with low NLR, indicating the profound prognostic impact of inflammatory imbalance in this population **[51]**.

In a cohort of 225 patients with stage 3–5 chronic kidney disease (CKD) followed for an average of 39 months, elevated neutrophil-to-lymphocyte ratio (NLR) was found to be a significant predictor of both fatal and nonfatal cardiovascular events (CVE). NLR increased progressively with advancing CKD stage and was independently associated with endothelial dysfunction, as measured by flow-mediated dilation (FMD), regardless of hsCRP levels. A baseline NLR ≥ 3.76 predicted composite CVE with 80.3% sensitivity and 91.8% specificity, and patients with higher NLR had significantly reduced survival time. These findings suggest that NLR is a useful independent marker of cardiovascular risk in moderate to severe CKD **[52]**.

Recent work by Carollo and colleagues, further contributed to the field by examining the prognostic value of NLR in a cohort of 236 chronic hemodialysis patients. Their findings confirmed that patients in the highest tertile of NLR not only had worse nutritional and iron parameters, but also significantly higher mortality risk. Interestingly, NLR demonstrated superior prognostic accuracy compared to C-reactive protein (CRP), with a higher area under the curve in ROC analysis, supporting its potential as a more sensitive marker of chronic inflammation in dialysis settings **[53]**.

Emerging evidence supports the association between increased NLR and accelerated decline in renal function, underscoring the role of inflammation in CKD progression. This pro-inflammatory environment not only contributes to renal parenchymal injury but also interferes with erythropoiesis, leading to reduced responsiveness to erythropoietin (EPO) therapy **[54]**. Resistance to EPO, characterized by a suboptimal hematologic response to ESAs, correlates with higher inflammatory burden as indicated by NLR elevation **[55]**. The mechanistic pathways involve inflammatory cytokines such as interleukin-6 and tumor necrosis factor-alpha, which inhibit erythroid progenitor proliferation and disrupt iron homeostasis, thereby attenuating EPO efficacy **[56]**.

Notably, EPO resistance serves as an independent predictor of adverse renal outcomes, with patients exhibiting both high NLR and EPO resistance experiencing more rapid CKD progression and increased cardiovascular morbidity **[57]**. Thus, integrating NLR assessment with evaluation of ESA responsiveness may enhance risk stratification and guide therapeutic strategies in CKD.

In conclusion, the interplay between systemic inflammation, measured by NLR, and erythropoietin resistance constitutes a critical axis influencing CKD progression. Targeted anti-inflammatory interventions aimed at improving EPO responsiveness hold promise in mitigating disease advancement and improving clinical outcomes.

Regarding CV and all-cause mortality in CKD patients, while it is well known that inflammation is common in CKD, it is less clear how well certain inflammatory markers can predict long-term survival in these patients.

To explore this, researchers analyzed data from nearly 7000 CKD patients collected over almost 20 years from the National Health and Nutrition Examination Survey (NHANES). They looked at four blood-based inflammatory indicators: the systemic immune-inflammation index (SII), NLR, PLR and LMR **[58]**.

The findings showed that higher levels of SII, NLR, and PLR were linked to a greater risk of death, whereas higher LMR levels appeared to have a protective effect. Among these, NLR and LMR stood out as the strongest predictors of survival in CKD patients. This suggests that measuring these markers could help doctors better identify patients who are at higher risk of poor outcomes.

Moreover, the relationship between these inflammatory markers and mortality risk was not straightforward, indicating that changes in inflammation levels may affect survival in complex ways. Catabay and colleagues investigated whether NLR and PLR, could help predict mortality in patients starting hemodialysis **[59]**. Among over 108,000 patients in a large U.S. dialysis network (2007–2011), NLR was significantly associated with increased mortality, particularly when measured over time. PLR showed a J-shaped association but did not significantly improve mortality prediction models.

NLR modestly improved predictive models beyond standard clinical parameters (like demographics, comorbidities, and serum albumin), as shown by improvements in AUROC, net reclassification index (NRI), and adjusted R^2^. In contrast, PLR provided little to no added predictive value.

From a translational standpoint, NLR represents more than a passive biomarker. Its integration into clinical risk models could enhance the precision of prognostication in CKD, particularly when combined with other clinical, biochemical, and possibly genomic indicators. Despite this promise, several critical knowledge gaps remain. First, direct comparisons between NLR and other inflammatory markers—such as (IL-6, tumor necrosis factor-alpha (TNF-α), or high-sensitivity C-reactive protein (hsCRP)—are limited. While such comparisons have been extensively explored in the context of acute inflammatory states, particularly COVID-19 **[60,61]**, similar head-to-head evaluations are scarce in the setting of chronic kidney disease. In nephrology, most comparative studies involving NLR are conducted against other blood cell–derived inflammatory indices, such PLR, MLR, or the composite lymphocyte-to-C-reactive protein ratio (LCR), rather than against canonical cytokine-based inflammatory markers. This limits our understanding of NLR’s relative performance and mechanistic specificity in capturing the complex inflammatory milieu characteristic of progressive renal dysfunction.

Secondly, the majority of current research on this topic is based on observational studies, and there remains a lack of interventional trials specifically targeting pathways related to NLR modulation, such as through anti-inflammatory or immunomodulatory treatments. To address this gap, findings from five recent randomized controlled trials—CANTOS, JUPITER, SPIRE-1, SPIRE-2, and CIRT—have been examined. These studies evaluated the effects of canakinumab, rosuvastatin, bococizumab, and methotrexate compared to placebo in patients with atherosclerosis. The data indicated that lipid-lowering agents did not produce meaningful changes in NLR levels. In contrast, treatment with canakinumab, an interleukin-1β–targeting monoclonal antibody, resulted in a significant reduction in NLR, suggesting a distinct effect of anti-inflammatory therapies on this biomarker. These observations highlight the need for further research into the influence of other immunomodulatory strategies, particularly those targeting interleukin pathways, on NLR dynamics **[62]**. Finally, population-based studies in healthy individuals are needed to better define normal reference ranges and thresholds for pathological significance.

Despite the growing evidence supporting the role of NLR as a prognostic marker in CKD, several studies have reported no significant association between NLR and renal disease progression or related clinical outcomes. For instance, in a retrospective 5-year study of 740 CKD stage 2–4 patients from Altunoren and colleagues, higher baseline NLR was associated with greater inflammation and faster GFR decline, but NLR was not an independent predictor of CKD progression. Instead, diabetes, younger age, and lower baseline eGFR were stronger predictors of reaching end-stage **[63]**. Similar results were reported by Yuan and colleagues, who investigated the association between NLR and progression to ESRD, cardiovascular events, and all-cause mortality in Chinese patients with stages 1–4 CKD. Their multicenter study demonstrated that NLR was associated with the risk of ESRD only in patients with stage 4 CKD, while no significant associations were observed between NLR and the risk of cardiovascular events or mortality in the overall cohort or within CKD stage subgroups **[64]**.

These discrepant results may be attributable to several factors, including heterogeneity in study designs, differences in patient populations, and the multifactorial nature of inflammation in CKD. In particular, the inflammatory state in advanced CKD and dialysis patients can be influenced by comorbid conditions, infections, medication use, and dialysis-related factors, which may confound NLR measurements and diminish its specificity as a biomarker for renal disease progression. Consequently, while NLR remains a promising and accessible marker of systemic inflammation, its role as a reliable prognostic indicator in CKD progression requires further validation through well-designed longitudinal studies accounting for these confounders.

In summary, NLR encapsulates the inflammatory burden of CKD and offers independent prognostic value for disease progression and cardiovascular morbidity. As inflammation becomes an increasingly recognized therapeutic target in nephrology, NLR may serve both as a stratification tool and a surrogate endpoint in future clinical trials. Its routine implementation in nephrology practice will require robust validation, standardization of thresholds, and integration into multifactorial predictive models to support personalized risk assessment and tailored therapeutic strategies. Table 2 summarizes the main studies reviewed in this section.

## 6. NLR and Cardiovascular Disease

The relationship between the NLR and cardiovascular disease represents one of the most extensively investigated areas in the study of inflammation-based prognostic biomarkers. A growing body of evidence supports the role of NLR as a reliable, independent predictor of both acute and long-term cardiovascular outcomes, including major adverse cardiovascular events (MACE), heart failure, arrhythmia, and cardiovascular mortality.

Large-scale meta-analyses and cohort studies have demonstrated that elevated NLR levels are consistently associated with worse prognosis in patients with acute coronary syndromes (ACS), particularly in ST-elevation myocardial infarction (STEMI) treated with percutaneous coronary intervention (PCI). In a meta-analysis of over 10,000 STEMI patients, higher admission NLR was linked to a markedly increased risk of in-hospital and long-term mortality (RR ≈ 3.3), MACE (RR ≈ 2.0), stent thrombosis (RR ≈ 2.7), and heart failure [65].

Similarly, in patients with non-ST-elevation myocardial infarction (NSTEMI), elevated NLR was shown to enhance long-term prognostic stratification. In a cohort study involving 678 NSTEMI patients undergoing PCI, combined elevation of NLR and MLR provided greater predictive accuracy for MACE than either marker alone [66].

Finally in another meta-analysis evaluated the prognostic utility of NLR in patients with ST-segment elevation myocardial infarction (STEMI) undergoing percutaneous coronary intervention (PCI), NLR, a marker of systemic inflammation, has been increasingly recognized as a potential predictor of adverse outcomes in cardiovascular disease. The primary objective of this study was to assess whether elevated NLR is associated with increased in-hospital and long-term mortality and major cardiovascular events in this high-risk patient population.

The pooled analysis demonstrated that higher baseline NLR was significantly associated with increased in-hospital all-cause mortality, cardiovascular mortality, and MACE. Moreover, elevated NLR was correlated with higher long-term mortality and MACE, as well as prolonged hospital stay. The association between elevated NLR and long-term cardiovascular mortality appeared particularly strong. These findings remained consistent across studies and statistical models, supporting the robustness of the association [67].

Caimi et al. investigated the neutrophil-to-lymphocyte ratio (NLR) in a cohort of 123 young patients (mean age 39.4 ± 5.8 years) with acute myocardial infarction (AMI), assessing values at the initial stage and at 3 and 12 months post-event [68]. They found that NLR was significantly elevated in young AMI patients compared to healthy controls (2.38 ± 0.87 vs. 1.82 ± 0.71, *p* < 0.0001). Notably, NLR did not differ significantly between STEMI and non-STEMI patients, nor between diabetic and non-diabetic individuals. However, smokers exhibited lower NLR values compared to non-smokers. Initial NLR levels were not correlated with the number of cardiovascular risk factors or the extent of coronary artery disease. Over time, a significant reduction in neutrophil count led to decreased NLR at both 3 and 12 months post-AMI, while lymphocyte counts remained stable. The same group observed in young patients with acute myocardial infarction both NLR and plasma viscosity (PV) increased during the acute phase, but their trajectories diverged over time. NLR significantly decreased at 3 and 12 months follow-up, while PV remained persistently elevated, suggesting different underlying mechanisms. The sustained rise in PV, unlike NLR, appeared linked to genetic predisposition and was associated with cardiovascular complications during follow-up [69].

The prognostic significance of NLR extends beyond ischemic heart disease. In patients hospitalized for acute decompensated heart failure, elevated NLR was independently associated with increased in-hospital mortality (OR ≈ 2.2) and three-year post-discharge mortality (OR ≈ 1.44), even after adjusting for age, renal function, and ejection fraction [70].

Moreover, in patients presenting with cardiogenic shock following acute MI, NLR > 7.3 at admission predicted a significantly higher 30-day mortality rate (73.7% vs. 26.3%) and was confirmed as an independent predictor of both all-cause death and MACE (HR ≈ 2.8) [71].

The pathophysiological rationale for these associations lies in the dual roles of neutrophils and lymphocytes during cardiovascular injury. Neutrophils exacerbate myocardial and vascular damage via the release of reactive oxygen species, proteolytic enzymes, and inflammatory mediators. Lymphocytes, on the other hand, are essential for resolving inflammation and facilitating tissue repair. Thus, an elevated NLR reflects a systemic inflammatory imbalance, skewed toward injury and inadequate repair—contributing to plaque instability, thrombosis, and maladaptive remodeling [72].

Beyond coronary syndromes and heart failure, elevated NLR has also shown prognostic relevance in atrial fibrillation (AF). A 2024 meta-analysis found that higher NLR levels were significantly associated with increased all-cause mortality (OR ≈ 1.87) and stroke (OR ≈ 1.56) in patients with AF, highlighting its broader cardiovascular implications [73].

Regarding atherosclerosis a large-scale study involving more than 100,000 participants examined the relationship between neutrophil levels and the risk of nine distinct cardiovascular outcomes using both observational and genetic methodologies. The findings indicated that elevated neutrophil counts were consistently linked to an increased risk across all atherosclerotic cardiovascular disease (ASCVD) endpoints, with comparable associations observed in both male and female subjects [74]. A retrospective study involving 4000 participants found that both the Chinese Visceral Adiposity Index (VAI) and NLR were independently associated with an increased risk of carotid atherosclerosis. Additionally, both markers demonstrated a significant positive correlation with the 10-year ASCVD (atherosclerotic cardiovascular disease) risk score, with all associations reaching strong statistical significance (*p* < 0.001) [75]. Wang et al. investigated the predictive value of the neutrophil-to-lymphocyte ratio (NLR) and plasma lipoprotein (a) [Lp (a)] levels for identifying unstable coronary plaques in a cohort of 1618 individuals with atherosclerotic cardiovascular disease (ASCVD), as assessed through coronary computed tomography angiography (CTA) [76]. In a similar context, several studies have recognized the neutrophil-to-lymphocyte ratio (NLR) as a promising and informative biomarker for detecting vulnerable carotid plaques [77], as demonstrated through carotid ultrasonography. These findings support the potential of NLR in assessing the likelihood of carotid plaque development and instability [78].

Collectively, these findings support the role of NLR as a readily available, cost-effective marker that could be integrated into risk stratification frameworks for patients with cardiovascular disease. However, further comparative studies are needed to assess its additive value over established markers such as high-sensitivity CRP, troponins, NT-proBNP, and interleukin-6. Table 3 summarizes the main studies reviewed in this section.

## 7. NLR and Hypertension

Hypertension is increasingly recognized not solely as a hemodynamic disorder but as a chronic, low-grade inflammatory condition. Sustained elevations in blood pressure induce mechanical stress on the vascular wall, promoting endothelial dysfunction, oxidative stress, and immune activation. These processes initiate and perpetuate a pro-inflammatory milieu characterized by increased circulating levels of interleukin (IL)-1β, IL-6, and tumor necrosis factor-alpha (TNF-α), which contribute to arterial remodeling, vascular stiffening, and progressive target organ damage [4,31,79].

A growing body of evidence supports the role of the NLR as a surrogate marker of systemic inflammation in hypertensive populations. Multiple epidemiological studies and meta-analyses have consistently demonstrated significantly higher NLR levels in hypertensive individuals compared to normotensive controls. This difference is particularly pronounced in “non-dipper” hypertensive patients, whose nocturnal blood pressure fails to decline physiologically—a pattern associated with greater cardiovascular risk and subclinical organ damage [80]. In a resistant hypertension cohort, NLR remained substantially elevated even after adjusting for confounders and showed a positive correlation with both office and ambulatory systolic and diastolic blood pressure values [81].

NLR has also been independently associated with left ventricular hypertrophy (LVH) in hypertensive patients. Yu et al. found that higher NLR levels were significantly linked to LVH (odds ratio = 1.506; 95% CI: 1.086–2.089; *p* = 0.014), with an optimal diagnostic cutoff of 2.185 (AUC = 0.626), comparable to CRP (AUC = 0.639) and BNP (AUC = 0.645) in terms of diagnostic performance [82].

The predictive value of NLR for the development of hypertension has also been demonstrated. In a prospective cohort study of 28,850 initially normotensive individuals, those in the highest NLR quintile had a significantly increased risk of developing hypertension over approximately six years (HR = 1.23; 95% CI: 1.06–1.43; *p* for trend < 0.01) compared to the lowest quintile, even after full adjustment [83].

A 2021 meta-analysis of 21 studies confirmed that hypertensive individuals had significantly higher NLR levels than normotensive controls (WMD = 0.40; 95% CI: 0.22–0.57; *p* < 0.0001). NLR was also significantly higher in non-dipper versus dipper hypertensive patients (WMD = 0.58; 95% CI: 0.19–0.97; *p* = 0.003), suggesting a potential link between blood pressure patterns, systemic inflammation, and cardiovascular risk [31].

A cross-sectional NHANES analysis (1999–2010; n = 22,290) further supported the association between NLR and hypertension prevalence. Per natural log-unit increase in NLR, the odds of hypertension increased (OR = 1.087; 95% CI: 1.008–1.173). Individuals in the highest NLR tertile had a 1.11-fold higher risk of hypertension compared to those in the lowest. While the systemic immune-inflammation index (SII) demonstrated even stronger associations, other markers such as platelet-to-lymphocyte ratio (PLR) and lymphocyte-to-monocyte ratio (LMR) were not significantly associated with hypertension [84].

In a large cohort of 6278 Taiwanese adults, higher NLR levels were significantly associated with prevalent hypertension. After adjusting for covariates, individuals in the highest NLR tertile had a 28% higher risk of hypertension (HR = 1.28; 95% CI: 1.03–1.59), with particularly strong associations in older adults (HR = 1.88; 95% CI: 1.19–2.96) and men over 60 years (HR = 1.84; 95% CI: 1.06–3.18), indicating the relevance of inflammatory markers in age-related hypertension risk [85].

The relationship between NLR and target organ damage has also been extensively investigated. In patients with resistant hypertension, NLR positively correlated with systolic and diastolic blood pressure measures, suggesting a direct link with vascular stress and structural adaptation. In one study of 150 individuals, NLR levels were significantly higher in resistant hypertension (RHT) compared to controlled hypertensives (CHT) (*p* = 0.03) and normotensives (NT) (*p* < 0.001). Multivariate analysis confirmed both NLR and absolute neutrophil count as independent predictors of RHT (*p* < 0.001), reinforcing the hypothesis of inflammatory involvement in blood pressure resistance [81].

In patients with H-type hypertension, characterized by elevated homocysteine levels, each unit increase in NLR was associated with a 51% higher risk of renal dysfunction, defined by reductions in eGFR and elevations in blood urea nitrogen and serum creatinine [86].

Further supporting its vascular relevance, in a study of 217 hypertensive patients and 132 normotensive controls, NLR levels were significantly higher in individuals with isolated systolic hypertension and combined systolic-diastolic hypertension. Brachial-ankle pulse wave velocity (baPWV), a surrogate marker of arterial stiffness, was elevated across all hypertensive subtypes and positively correlated with NLR. Multivariate analysis confirmed NLR as an independent predictor of baPWV, suggesting that systemic inflammation—as indexed by NLR—may actively contribute to arterial stiffening and atherosclerotic changes [87].

Importantly, NLR also has prognostic value. In a cohort of 3067 hypertensive adults from NHANES (2009–2014), an elevated NLR (>3.5) was independently associated with increased all-cause mortality (HR = 1.96; 95% CI: 1.52–2.52) and cardiovascular mortality (HR = 2.33; 95% CI: 1.54–3.51) over a median follow-up of 92 months. A clear dose–response relationship was observed, and eGFR partially mediated this association (5.4% for all-cause and 4.7% for cardiovascular mortality). Time-dependent AUCs for NLR predicting 3-, 5-, and 10-year survival ranged from 0.64 to 0.70, underscoring its moderate prognostic value [88].

These findings were subsequently validated in a larger cohort using a lower NLR threshold (>2.0), which remained significantly associated with both all-cause (HR ≈ 1.47) and cardiovascular mortality (HR ≈ 2.08) [89].

Overall, NLR emerges as a robust, inexpensive, and readily accessible biomarker that integrates the inflammatory dimension of hypertension. Its consistent associations with hypertension incidence, blood pressure patterns, target organ damage, vascular stiffness, and long-term mortality make it a promising candidate for clinical risk stratification and potentially for guiding anti-inflammatory strategies in hypertension management. However, despite its growing relevance, direct comparative studies between NLR and other established or emerging inflammatory biomarkers—such as high-sensitivity CRP, interleukin-6, or the systemic immune-inflammation index (SII)—remain limited. Future research is needed to evaluate the relative diagnostic, prognostic, and therapeutic value of NLR in comparison to these markers, particularly in diverse hypertensive subpopulations. Such data will be essential to clarify whether NLR should be adopted as a first-line inflammatory marker or used in conjunction with a broader panel in clinical practice. Table 4 summarises the main studied reviewed in this section.

## 8. Current Limitations and Gaps

Current limitations and gaps in the literature underscore the need for further standardization and validation of inflammatory markers such as the neutrophil-to-lymphocyte ratio (NLR) in clinical practice. Notably, there is a lack of universally accepted and standardized cut-off values for NLR, both in healthy populations and in patients with chronic diseases, which hampers its broader clinical implementation. Furthermore, data on NLR distribution in healthy individuals are limited, and population profiling studies aimed at establishing normative reference ranges are still lacking. For instance, diurnal variation has been reported, with neutrophil counts peaking in the morning and lymphocyte counts being relatively higher later in the day, leading to fluctuating NLR [90]. In addition, acute infections, surgery, trauma, and psychological or physical stress can transiently elevate NLR, reflecting short-term immune activation rather than chronic low-grade inflammation. Pharmacological interventions, including corticosteroids, immunosuppressive agents, or chemotherapy, also alter leukocyte subpopulations and therefore affect NLR independently of disease activity [14]. Such sources of variability should be acknowledged when using NLR for risk stratification, particularly in longitudinal or comparative studies.

Most available evidence derives from retrospective or single-center studies, with a particular scarcity of large-scale, prospective, multicenter investigations—especially in populations with diabetes mellitus.

Although this is a narrative review, we have considered the methodological quality of the available studies. Most of the evidence derives from observational studies with heterogeneous populations and variable adjustment for confounding factors. Only a limited number of randomized clinical trials are available, which reduces the strength of causal inferences. Nevertheless, the consistency of findings across diverse clinical settings supports the robustness of the association between NLR and cardio-renal-metabolic disorders. Moreover, NLR is not currently integrated into established composite cardiovascular risk models, such as SCORE or the Framingham Risk Score, limiting its utility in risk stratification. Defining clinically meaningful thresholds tailored to high-risk subgroups (e.g., patients with chronic kidney disease, diabetes, or established cardiovascular disease) is essential. Additionally, no interventional studies have yet assessed whether targeting NLR can improve clinical outcomes. Preliminary evidence suggests that integrating NLR with other biomarkers—such as high-sensitivity C-reactive protein (hs-CRP) or troponins—may enhance predictive accuracy, warranting further investigation.

## 9. Future Perspectives and Conclusions

The neutrophil-to-lymphocyte ratio (NLR) has emerged as a simple yet powerful inflammatory biomarker with broad potential applications in cardiovascular, renal, and metabolic medicine. Given its accessibility, cost-effectiveness, and robust association with adverse outcomes in hypertension, chronic kidney disease (CKD), and diabetes, NLR represents a promising candidate for integration into personalized medicine frameworks.

In the context of personalized risk stratification, NLR may serve as a valuable tool for identifying individuals at heightened risk of adverse events, particularly in those with overlapping cardio-metabolic comorbidities. When combined with clinical parameters—such as blood pressure profiles, renal function indices, and glycemic markers—and genetic or epigenetic signatures, NLR could enhance secondary prevention strategies, allowing for earlier and more targeted interventions.

Moreover, the incorporation of NLR into risk prediction algorithms and clinical decision-support systems may facilitate refined triage and monitoring protocols. Its dynamic responsiveness to inflammatory shifts suggests utility in assessing therapeutic efficacy, particularly in settings of resistant hypertension, diabetic nephropathy, or cardiorenal syndromes. The potential to track longitudinal changes in NLR could help guide medication adjustments, lifestyle interventions, or escalation of care in high-risk individuals.

Despite its promise, several gaps remain. Notably, direct comparative studies between NLR and other validated inflammatory or prognostic biomarkers—such as high-sensitivity C-reactive protein (hsCRP), interleukin-6, or novel immune-metabolic indices—are lacking, especially on a disease-specific basis. Such comparisons are essential to contextualize the unique value of NLR in clinical decision-making. Table 5 provides a comparison of NLR and other inflammatory markers’ prognostic role.

Additionally, normative data for NLR in healthy populations across age, sex, and ethnicity are sparse. Establishing reference ranges and population-specific thresholds is crucial for distinguishing pathologic inflammation from physiological variability, particularly in asymptomatic or early-stage patients.

Ultimately, realizing the full clinical utility of NLR will depend on translational research efforts and well-designed prospective validation studies. These should aim to define cut-off values, evaluate predictive performance in diverse clinical settings, and explore integration with other biomarkers in multi-parametric approaches. As the field moves toward precision cardiometabolic care, NLR holds significant potential—but its adoption must be guided by rigorous scientific validation.

## Figures and Tables

**Table 1:** Summary of the main studies reviewed, highlighting key characteristics such as study design, sample size, population, methodology, and main findings.

Author, Year	Population	Main Outcomes	Key Findings
**Li et al., 2019 [43]**	3000+ T2DM patients	NLR correlation with renal function	NLR significantly higher in patients with diabetic nephropathy
**Adane et al, 2023 [44]**	13 studies meta-analysis	Relationship between NLR and glycemic control	NLR positively correlated with HbA1c and poor glycemic control
**Rui-Wang et al., 2020 [46]**	470 T2DM and controls	Association between NLR, PLR and DR	NLR independently associated with diabetic retinopathy as continuous and categorical variable
**Si et al. 2024 [45]**	572 adults with diabetic retinopathy (NHANES 2009–2018)	All-cause mortality, diabetes-related cardiovascular mortality	Elevated NLR (≥1.516), MLR (≥0.309), and SIRI (≥0.756) independently associated with increased mortality risk. Combined marker (NLR + MLR + SIRI) improved prognostic performance. J-shaped mortality curves observed.

**Table 2:** Summary of the main studies reviewed, highlighting key characteristics such as study design, sample size, population, methodology, and main findings.

Study (Author, Year)	Population	Main Outcomes	Key Findings
**Yoshitomi et al., 2019 [47]**	256 CKD stage 3–5, Japan	eGFR decline, renal replacement therapy	Higher NLR associated with faster renal decline and dialysis initiation
**Wang et al., 2021 [48]**	966 IgA nephropathy patients	ESKD progression, histological severity	NLR ≥ 2.67 linked to glomerulosclerosis and fibrosis, higher progression risk
**Muresan et al., 2023 [51]**	ESKD Romanian patients (2016–2019)	NLR/MLR/PLR at admission vs. 30-day mortality	High NLR: 30-day mortality = 40.12% vs. 1.97% (*p* < 0.0001); independent predictor of early death
**Yuan et al., 2021 [64]**	938 Chinese CKD patients, stages 1–4	ESKD progression, CV and all-cause mortality	NLR associated with ESKD risk only in stage 4 CKD; no significant association with CVD events or all-cause mortality across all stages
**Chen et al., 2024 [58]**	3500 NHANES CKD participants	All-cause mortality	Elevated NLR associated with higher 10-year mortality (HR > 1.5)
**Carollo et al. 2025 [53]**	236 chronic hemodialysis patients	12-month mortality, inflammation/nutrition markers	High NLR linked to increased mortality and worse nutritional profile
**Lin et al., 2021 [49]**	3000+ patients, Taiwan	eGFR decline, albuminuria, inflammation	Higher NLR linked to progression in both diabetic and non-diabetic CKD
**Ao, et al., 2021 [50]**	116,709 patients with CKD, including those on dialysis (13 studies)	CV and all-cause mortality	Elevated NLR was significantly associated with increased all-cause mortality (HR 1.93) and cardiovascular mortality (HR 1.45); among dialysis patients, all-cause mortality HR 1.94
**Catabay et al., 2017 [59]**	Incident HD patients (2007–2011, USA)	Short- & long-term mortality	High NLR predicted mortality; improved AUROC and R^2^; PLR had no predictive value

**Table 3:** Summary of the main studies reviewed, highlighting key characteristics such as study design, sample size, population, methodology, and main findings.

Setting/Study	Design & Sample	NLR Cut-Off/Quartile	Outcome(s)	Key Prognostic Findings
**Ul Hussain et al. 2024 [67]**	STEMI patients post-PCI; 28,756 individuals (35 studies)	High vs. low NLR (varied)	In-hospital & long-term mortality, CV mortality, MACE	High NLR: in-hospital mortality RR = 3.52; long-term mortality HR ≈ 1.07; CV mortality RR ≈ 2.66; MACE RR ≈ 2.92
**Cho et al., 2018 [70]**	Acute HF	Elevated NLR (thresholds unspecified)	In-hospital mortality, three years mortality	Elevated NLR independently associated with increased in-hospital mortality (OR ≈ 2.2) and three-year post-discharge mortality (OR ≈ 1.44)
**Caimi et al. 2024 [68]**	123 young AMI patients (mean age 39.4 ± 5.8 years)	NLR ≥ 6.5	STEMI/Non STEMI Long term outcome	NLR significantly elevated in AMI patients vs. controls (2.38 ± 0.87 vs. 1.82 ± 0.71, *p* < 0.0001). No difference in NLR between STEMI vs. non-STEMI or diabetic vs. non-diabetic. NLR not correlated with number of CV risk factors or CAD extent. Neutrophil count decreased over time leading to reduced NLR; lymphocyte counts stable.
**Peng et al. 2024 [73]**	AF cohort meta-analysis; ~59,000 patients	High NLR (varied)	AF recurrence, stroke, mortality, left atrial thrombus	High NLR predicted AF recurrence and stroke; linked to mortality and thrombus
**Cho et al., 2020 [70]**	Decompensated HF; 5580 patients	Quartile 4 (not specified)	In-hospital & 3-year mortality	Highest quartile NLR: OR ~2.2 for in-hospital mortality; OR ~1.44 at 3 years

**Table 4:** Summary of the main studies reviewed, highlighting key characteristics such as study design, sample size, population, methodology, and main findings.

Study (Author, Year)	Design/Population	Main Focus/Outcome	Key Findings
**Zhang et al., 2024** **[88]**	NHANES 2009–2014; 3067 hypertensive adults	NLR and mortality	NLR > 3.5 associated with higher all-cause (HR = 1.96) and CV mortality (HR = 2.33); AUC 0.64–0.70
**Hong et al., 2024 [89]**	NHANES (extended sample)	NLR thresholds and mortality	NLR > 2.0 linked to all-cause (HR~1.47) and CV mortality (HR~2.08)
**Liu et al., 2023** **[83]**	Prospective cohort; 28,850 normotensives	Incident hypertension	Highest NLR quintile: HR = 1.23 for new-onset hypertension
**Sarejiloo et al., 2021 [31]**	21 studies	NLR in hypertensive vs. normotensive	WMD = 0.40; Non-dippers showed higher NLR (WMD = 0.58)
**Yu et al., 2020 [82]**	Cross-sectional; hypertensive patients	NLR and LVH	NLR independently associated with LVH (OR = 1.506); AUC = 0.626
**Wang et al., 2017 [87]**	217 hypertensive + 132 controls	NLR and arterial stiffness (baPWV)	NLR higher in ISH and SDH; NLR independently predicted baPWV
**Belen et al. [81]**	Cohort of resistant hypertension	NLR and BP severity	NLR correlated with office and ambulatory BP; elevated in RHT
**Chen et al. [86]**	H-type hypertension patients	NLR and renal function	1 unit ↑ NLR = 51% ↑ risk of renal dysfunction
**NHANES 1999–2010 [84]**	Cross-sectional; n = 22,290	NLR and HTN prevalence	ln-NLR: OR = 1.087 for HTN; SII stronger; PLR and LMR not significant
**Jhuang et al., 2019 [85]**	6278 adults	NLR and prevalent hypertension	HR = 1.28 overall; stronger in older adults (HR = 1.88)
**Sunbul et al., 2014 [80]**	83 Non-dipper hypertensives	NLR and BP pattern	NLR > 2.7 predicted non-dipping (83% sens., 65% spec.)

ISH: Isolated Systolic Hypertension; SDH: Systolic-Diastolic Hypertension; LVH: Left Ventricular Hypertrophy; baPWV: Brachial-Ankle Pulse Wave Velocity; CV: Cardiovascular.

**Table 5:** Comparison of NLR and other inflammatory/prognostic biomarkers.

Biomarker	Biological Significance	Biological Variability	Advantages	Limitations	Main Prognostic Evidence
**NLR (Neutrophil-to-Lymphocyte Ratio)**	Ratio between neutrophils (innate inflammation) and lymphocytes (adaptive response)	Influenced by circadian rhythm, infections, stress, corticosteroids, other therapies	Simple, inexpensive, derived from standard blood count; reflects immune balance	Non-specific, intra-individual variability; lack of standardized cut-off values	Associated with prognosis in sepsis, cardiovascular diseases, cancer, COVID-19
**CRP (C-reactive protein)**	Acute-phase protein produced by the liver in response to IL-6	Relatively stable; rises within 6–8 h after inflammatory stimulus	Widely validated; available in labs and point-of-care; standardized marker	Cannot differentiate acute vs. chronic inflammation; less informative on immune status	Strong marker of acute inflammatory severity and cardiovascular risk
**IL-6**	Central pro-inflammatory cytokine, stimulates CRP production	High intra-individual variability; short half-life; rapid peaks	Sensitive marker of early inflammatory activation	Requires dedicated assays; costly; less accessible in routine clinical use	Strongly associated with severity in sepsis, ARDS, COVID-19
**TNF-α**	Key cytokine in systemic inflammation and tumor necrosis	High variability; low basal levels; transient peaks	Early indicator of systemic inflammation	Measurement complex, less standardized; high cost	Involved in sepsis and autoimmune diseases; prognostic potential but less established compared with CRP/IL-6
